# An examination of global research trends for exploring the associations between the gut microbiota and nonalcoholic fatty liver disease through bibliometric and visualization analysis

**DOI:** 10.1186/s13099-024-00624-w

**Published:** 2024-07-03

**Authors:** Sa’ed H. Zyoud, Samer O. Alalalmeh, Omar E. Hegazi, Muna Shakhshir, Faris Abushamma, Samah W. Al-Jabi

**Affiliations:** 1 Poison Control and Drug Information Center (PCDIC), College of Medicine and Health Sciences, An-Najah National University, Nablus, 44839 Palestine; 2 Department of Clinical and Community Pharmacy, College of Medicine and Health Sciences, An-Najah National University, Nablus, 44839 Palestine; 3 Clinical Research Centre, An-Najah National University Hospital, Nablus, 44839 Palestine; 4 College of Pharmacy and Health Sciences, Ajman University, Ajman, United Arab Emirates; 5 Department of Nutrition, An-Najah National University Hospital, Nablus, 44839 Palestine; 6 Department of Medicine, College of Medicine and Health Sciences, An-Najah National University, Nablus, 44839 Palestine; 7 Department of Urology, An-Najah National University Hospital, Nablus, 44839 Palestine

**Keywords:** Nonalcoholic fatty liver disease, NAFLD, Gut microbiota, Bibliometric, Visualization

## Abstract

**Background:**

Nonalcoholic fatty liver disease (NAFLD) is increasingly recognized as a significant health issue. Emerging research has focused on the role of the gut microbiota in NAFLD, emphasizing the gut-liver axis. This study aimed to identify key research trends and guide future investigations in this evolving area.

**Methods:**

This bibliometric study utilized Scopus to analyze global research on the link between the gut microbiota and NAFLD. The method involved a search strategy focusing on relevant keywords in article titles, refined by including only peer-reviewed journal articles. The data analysis included bibliometric indicators such as publication counts and trends, which were visualized using VOSviewer software version 1.6.20 for network and co-occurrence analysis, highlighting key research clusters and emerging topics.

**Results:**

Among the 479 publications on the gut microbiota and NAFLD, the majority were original articles (*n* = 338; 70.56%), followed by reviews (*n* = 119; 24.84%). The annual publication count increased from 1 in 2010 to 118 in 2022, with a significant growth phase starting in 2017 (R^2^ = 0.9025, *p* < 0.001). The research was globally distributed and dominated by China (*n* = 231; 48.23%) and the United States (*n* = 90; 18.79%). The *University of California, San Diego*, led institutional contributions (*n* = 18; 3.76%). Funding was prominent, with 62.8% of the articles supported, especially by the *National Natural Science Foundation of China* (*n* = 118; 24.63%). The average citation count was 43.23, with an h-index of 70 and a citation range of 0 to 1058 per article. Research hotspots shifted their focus post-2020 toward the impact of high-fat diets on NAFLD incidence.

**Conclusions:**

This study has effectively mapped the growing body of research on the gut microbiota-NAFLD relationship, revealing a significant increase in publications since 2017. There is significant interest in gut microbiota and NAFLD research, mainly led by China and the United States, with diverse areas of focus. Recently, the field has moved toward exploring the interconnections among diet, lifestyle, and the gut-liver axis. We hypothesize that with advanced technologies, new opportunities for personalized medicine and a holistic understanding of NAFLD will emerge.

## Background

In recent decades, a significant number of health complications, including cardiovascular disorders, diabetes, obesity, and metabolic syndrome, have emerged as threats to public health [1–3]. Among these threats, nonalcoholic fatty liver disease (NAFLD) has also been recognized as a public health problem [4, 5]. The term NAFLD incorporates a range of liver disorders that collectively share the common feature of excess fat in the absence of significant alcohol consumption (from simple steatosis to nonalcoholic steatohepatitis) [4, 6]. NAFLD affects approximately 47 individuals out of every 1,000 individuals or more than 38% of the population and is associated with significantly increased all-cause mortality [7, 8].

Different risk factors are hypothesized to influence the development and progression of NAFLD. An accepted hypothesis is that insulin resistance, obesity, the production of reactive oxygen species, and genetics play a role in the pathogenesis of NAFLD. Specifically, insulin resistance contributes to fat accumulation in liver cells through increased triglycerides and fatty acids, along with reduced excretion and carbohydrate-induced fatty acid synthesis, leading to steatosis. Obesity is believed to aggravate liver damage by releasing inflammatory substances such as leptin from fat tissue, leading to liver cell destruction [6, 9, 10].

Moreover, evidence suggests that irregularities in the composition of intestinal microorganisms are additional risk factors for specific diseases [11–16]. The gut microbiota is known for its substantial impact on various bodily functions, including host metabolism, immune function, and even neurobehavioral traits [17–20]. The connection between the gut and the liver is commonly referred to as the “gut-liver axis” [12]. An alternative proposed mechanism involves dysfunction of the intestinal barrier, permitting gut bacterial metabolites to enter the liver, thereby increasing inflammation, oxidative stress, and lipid accumulation. This cascade expedites liver injury and fibrosis and influences the progression of NAFLD [20–22].

The discovery of this relationship and the subsequent growing interest in this relationship have led to a recognizable increase in the number of relevant scientific publications [23]. However, the sheer number, complexity of contexts, and diversity of publications make it challenging to synthesize and understand overarching trends and contributions in this field. This is where bibliometric analysis comes in [24, 25]. By assessing publication output, identifying research themes, and mapping collaborative networks, bibliometric analyses can help in identifying patterns and trends of publications in addition to quantifying contributions to the field and identifying future research directions.

Generally, systematic reviews, scoping reviews, and bibliometric analyses might appear similar due to their overlapping elements; however, they differ significantly. Bibliometric analyses are designed to perform a survey of literature on a specific subject. Unlike systematic reviews, which provide a targeted and specific assessment, bibliometric analyses offer a more holistic view [26]. While systematic reviews typically use a limited set of publications to address a precise research question, bibliometric analyses include a broader range of literature [26, 27]. Scoping reviews, on the other hand, focus on charting the scope and nature of the existing evidence to assist further in-depth studies [28]. In contrast, bibliometric analyses expand beyond this by providing publication frequency, authorship patterns, and citation analysis. These data enable an understanding of trends and patterns within a research area, making bibliometric analyses invaluable for identifying research gaps [29, 30].

Numerous studies have examined research trends within the realms of microbiota and the liver, encompassing a broad range of diseases [31–39]. Additionally, bibliometric analyses have investigated the gut microbiota independently [25, 40–43], as well as the incidence of NAFLD [44–47], with a notable absence of integration between these subjects. Therefore, this study intends to fill this gap by conducting a bibliometric analysis that combines both. The objective is to uncover trends and patterns in the research field, examine global and institutional collaborations, and determine key funding agencies. This study will also highlight noteworthy journals and articles in this area, in addition to providing future directions.

## Methods

### Study design

A descriptive, retrospective, cross-sectional, and bibliometric study design was used in this study.

### Database

Due to its comprehensive coverage and advanced search capabilities, the Scopus database was selected as the sole source for the evaluation of the global scientific output of the gut microbiota and its connection to NAFLD. In bibliometric research, utilizing a single database is customary because combining data from multiple sources can complicate bibliometric analyses and literature mapping. Additionally, gray literature, which encompasses non-peer-reviewed materials, cannot be effectively integrated into data retrieved from multiple databases [48–50].

The decision to use the SciVerse Scopus database for this study stemmed from several factors. First, Scopus boasts a far greater volume and diversity of indexed publications than its counterparts, PubMed and Web of Science. In fact, Scopus’s journal index nearly surpasses the combined index of PubMed and Web of Science [51–55]. Second, Scopus encompasses all the articles listed in PubMed, ensuring complete coverage of the PubMed literature within Scopus. As a result, Scopus is considered a comprehensive database that includes both PubMed and Web of Science publications [51–55]. Furthermore, Scopus’s interdisciplinary nature, spanning science, technology, medicine, social science, and the arts and humanities, aligns with the diverse range of research on the gut microbiota and NAFLD. Additionally, the advanced search functionality of Scopus, which employs various Boolean operators, facilitates the creation of sophisticated and comprehensive search queries. Finally, Scopus empowers researchers to seamlessly export and analyze retrieved data, enabling mapping and statistical analyses. Considering the rapid update cycle of the database, literature retrieval was conducted on a single day, November 22, 2023. Consequently, the study’s publication period encompassed the entire preceding year, up to December 31, 2022.

### Search strategies

Utilizing the “Advanced search” functionality of the Scopus online database, we used relevant keywords to identify literature pertaining to the gut microbiota and nonalcoholic fatty liver disease (NAFLD). The search strategy involved utilizing synonyms for both the gut microbiota and NAFLD as follows:

#### Step 1

First, we gathered terms related to the gut microbiota from prior research on the topic [24, 25, 41, 56–60] and medical subject headings (MeSH) from PubMed. These selected terms were subsequently included in the “Article Title” field of the Scopus search engine to accomplish the goals of our study: “Colonic flora” OR “Colonic microflora” OR “Colonic microbiome” OR “Colonic microbiota” OR “Digestive flora” OR “Digestive microflora” OR “Digestive microbiome” OR “Digestive microbiota” OR “Enteric bacteria” OR “Enteric flora” OR “Enteric microflora” OR “Enteric microbiome” OR “Enteric microbiota” OR “Fecal flora” OR “Fecal microflora” OR “Fecal microbiome” OR “Fecal microbiota” OR “Gastric flora” OR “Gastric microflora” OR “Gastric microbiome” OR “Gastric microbiota” OR “Gastrointestinal flora” OR “Gastrointestinal microflora” OR “Gastrointestinal microbiome” OR “Gastrointestinal microbiota” OR “Gastrointestinal microbial community” OR “Gastrointestinal microflora” OR “Gut flora” OR “Gut microflora” OR “Gut microbiome” OR “Gut microbiota” OR “Intestinal flora” OR “Intestinal microflora” OR “Intestinal microbiome” OR “Intestinal microbiota”.

#### Step 2

Following the initial step, we further refined our search by limiting the identified publications to those that included the terms “nonalcoholic fatty liver disease and associated terms” in their titles. The terms associated with NAFLD were sourced from the Medical Subject Headings (MeSH) in PubMed and subsequently entered into the Scopus database for this purpose. The following ‘terms’ were entered as ‘Article Title’: “NAFLD” OR “Non alcoholic Fatty Liver” OR “Non-alcoholic Fatty Liver” OR “Nonalcoholic Fatty Liver” OR “Nonalcoholic Steatohepatitis” OR “Non alcoholic Steatohepatitis” OR “Non-alcoholic Steatohepatitis”. In 2020, a new term was introduced for fatty liver disease: metabolic dysfunction-associated fatty liver disease (MAFLD). This name change reflects the understanding that this condition is linked to broader problems with metabolism throughout the body [61]. Our research focused on the well-defined term “NAFLD”, which was the established standard at the time of data collection. While we acknowledge the existence of the related term “MAFLD”, we opted to focus on NAFLD due to its wider recognition and established trends in research. Our decision reflects our specific interest in NAFLD itself rather than encompassing broader related terminology.

#### Step 3

The research limits its scope to peer-reviewed scientific journal articles, excluding books, book chapters, retracted articles, and errata.

### Validation of the search strategy

Two biomedical science colleagues, well versed in bibliometrics, were enlisted to validate the search strategy. This validation involved two distinct methods. Initially, the colleagues were tasked with ensuring the absence of false-positive articles by scrutinizing 47 randomly selected articles from the retrieved document (articles ranked 10, 20, 30, etc., based on citations). Valuable feedback from volunteers contributed to refining the research strategy. Subsequently, the experts were directed to compare the publication counts of the top 20 active authors with the actual number of articles for each scholar, examining their respective Scopus profiles. To ascertain the significance and correlation coefficient, the results from both methods were subjected to correlation testing. The results of the correlation test revealed a strong correlation coefficient (*r* = 0.987), and the statistical significance (*p* < 0.001) underscored the accuracy of the search query. This dual-method approach aimed to verify the absence of false-negative outcomes, drawing inspiration from previously published bibliometric studies [49, 62]. Notably, keywords were employed in the title search rather than in the title/abstract search, enhancing the reliability of the approach. Consequently, the title search emerged as a dependable method with minimal false-positive documents, unlike the title/abstract search [29, 48, 50], which yielded numerous false positives with a focus not specifically on NAFLD and the gut microbiota.

### Bibliometric analysis

Bibliometric indicators, including the total number of publications, publication years, types of publications, top ten funding agencies, top ten countries, top ten institutions, top ten journals, and the top ten most cited articles, were gathered using an Excel spreadsheet.

### Visualization analysis

The intricate connections between terms and collaborating countries were visualized using VOSviewer software version 1.6.20 (Leiden University, Leiden, The Netherlands). Network maps were constructed to depict the interplay of terms extracted from article titles or abstracts and the collaborative ties between countries [63–65]. A co-occurrence analysis was simultaneously performed to segregate terms into distinct clusters, which were further enhanced by color coding based on their temporal distribution. To assess the emergence of new topics and identify evolving trends, the average publication year was calculated.

## Results

### General description of the retrieved publications

This study included a total of 479 publications. Among them, articles constituted the majority, with 338 publications, composing 70.56% of the overall records and establishing them as the most prevalent literary form. A total of 119 publications were identified, accounting for 24.84% of the total. The remaining five types of publications, namely, documents such as letters, notes, editorials, minutes of meetings, and short surveys, totalled 22, representing 4.59% of the overall corpus.

### Growth and productivity trends

**Fig. 1:**
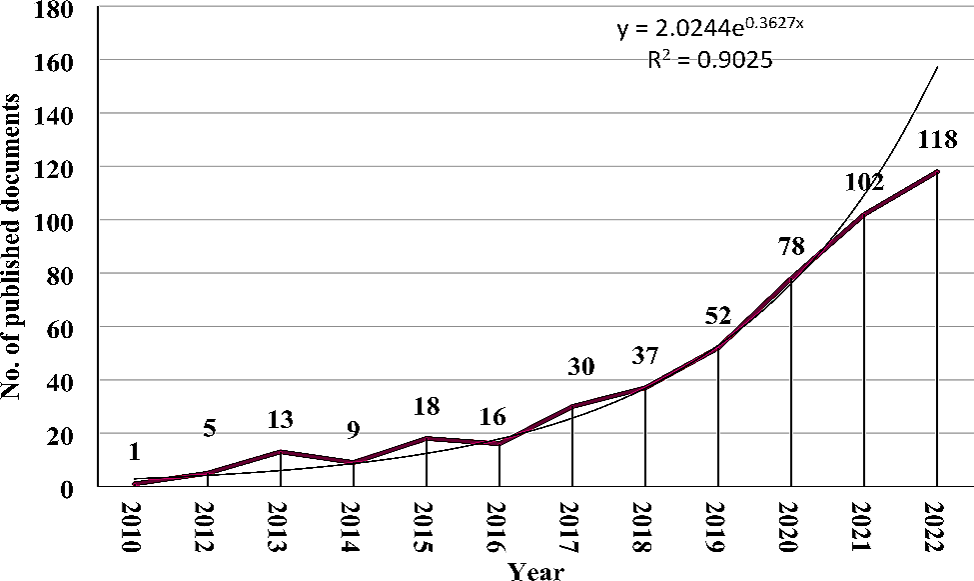
Growth trends in publications on gut microbiota and nonalcoholic fatty liver disease from 2010 to 2022

The number of publications on the gut microbiota and NAFLD increased each year during the study period. The number of publications ranged from 1 in 2010 to 118 in 2022, as shown in Fig. 1. Two distinct stages of growth were observed: the first stage, from 2010 to 2016, had a slow rate of publication production, while the second stage, from 2017 to 2022, had a much faster rate of publication progress. Statistical analysis using linear regression confirmed this observation and indicated a strong positive correlation (R^2^ = 0.9025, *p* < 0.001) between the annual publication count and the corresponding publication year.

### Top active countries

**Fig. 2:**
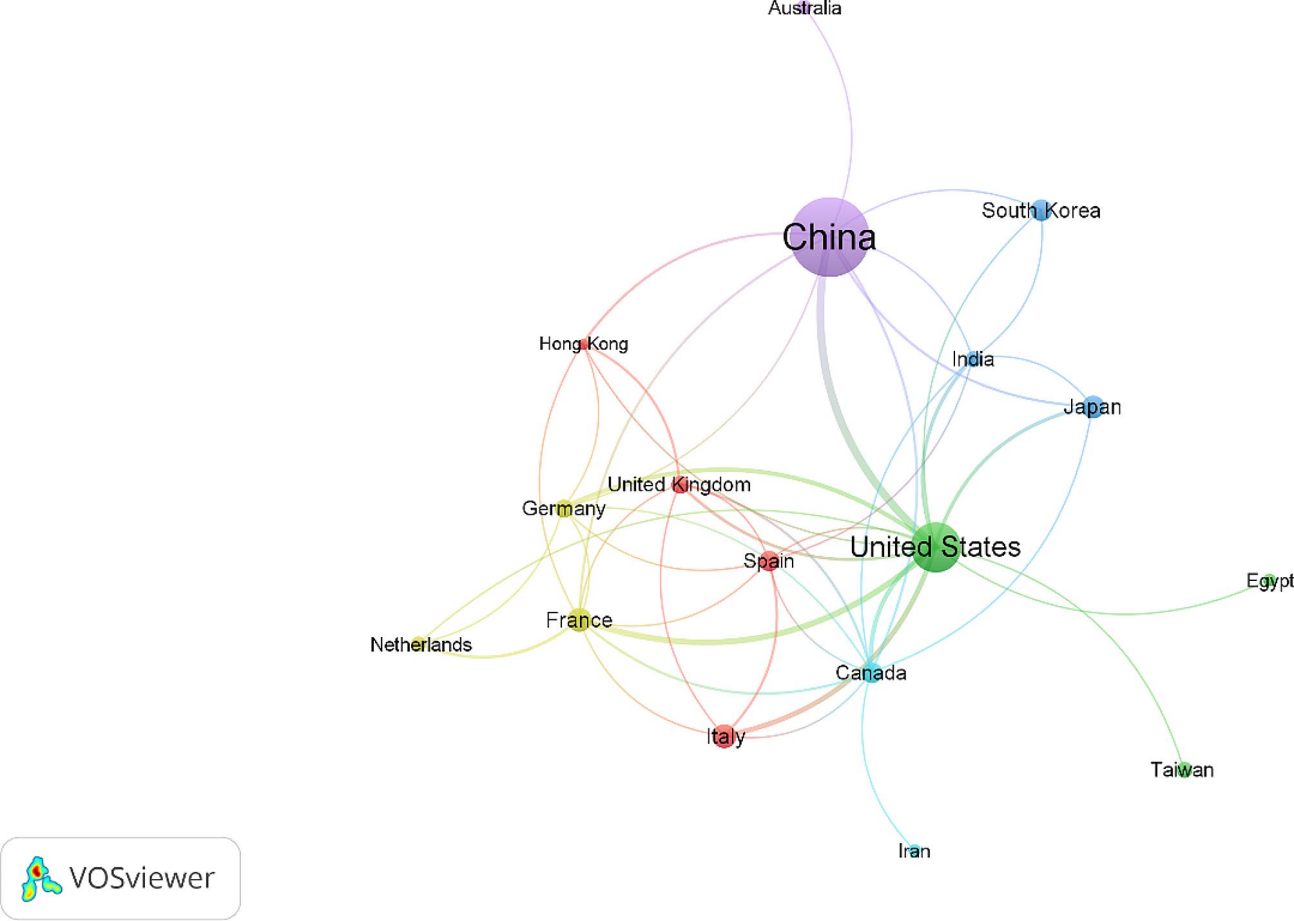
Visualization of international research collaboration networks related to the gut microbiota and NAFLD: 2010–2022. This collaborative network map showcases the interactions among leading countries engaged in research on the gut microbiota and nonalcoholic fatty liver disease from 2010 to 2022. The map is based on a threshold of at least 5 publications per country, with 21 out of the 47 active countries meeting this criterion. The size of each node on the map corresponds to the number of publications from that respective country. The map was generated using VOSviewer software version 1.6.20

Between 2010 and 2022, researchers conducted studies on the gut microbiota and NAFLD in a diverse array of 47 countries. In particular, the top ten countries represented a substantial 82.67% of all relevant research, as outlined in Table 1. China was the primary contributor, with 231 articles (48.23%), followed by the United States (90 articles; 18.79%), France and Italy, each contributing 22 articles (4.59%). Furthermore, the U.S. and China displayed significant involvement in international collaboration, leading to publications featuring scholars from various nations. To visually represent these global research networks, Fig. 2 illustrates a network mapping chart depicting international collaborations in studies on the gut microbiota and NAFLD among the prominent participating countries from 2010 to 2022.

**Table 1:** List of the top 10 countries publishing research on the gut microbiota and NAFLD from 2010 to 2022

Ranking	Country	Number of documents	%
1st	China	231	48.23
2nd	United States	90	18.79
3rd	France	22	4.59
3rd	Italy	22	4.59
5th	Japan	20	4.18
6th	South Korea	18	3.76
7th	Canada	15	3.13
7th	Spain	15	3.13
9th	Germany	13	2.71
9th	Ukraine	13	2.71

### Top active institutions

Table 2 provides a comprehensive list of the ten most productive institutes in the field of NAFLD and its correlation with the gut microbiota covering the period from 2010 to 2022. Together, these leading institutions contributed to 20.25% (*n* = 97) of the total articles published on the subject. The *University of California, San Diego*, emerged as the leading contributor globally, producing 18 publications and generating 3.76% of the overall publications. Similarly, *Shanghai University of Traditional Chinese Medicine* in China secured the second position with 17 publications (3.55%), followed by the *Ministry of Education of the People’s Republic of China* with 15 publications (3.13%) and the *National Academy of Medical Sciences of Ukraine* with 10 publications (2.09%) (Table 2).

**Table 2:** List of the top 10 institutions publishing research on the gut microbiota and nonalcoholic fatty liver disease from 2010 to 2022

Ranking	Institute	Country	*n*	%
1st	*University of California, San Diego*	USA	18	3.76
2nd	*Shanghai University of Traditional Chinese Medicine*	China	17	3.55
3rd	*Ministry of Education of the People’s Republic of China*	China	15	3.13
4th	*National Academy of Medical Sciences of Ukraine*	Ukraine	10	2.09
5th	*Instituto de Salud Carlos III*	Spain	9	1.88
5th	*Southern Medical University*	China	9	1.88
5th	*Shanghai Jiao Tong University*	China	9	1.88
5th	*Zhejiang University School of Medicine*	China	9	1.88
9th	*Centro de Investigación Biomédica en Red de Enfermedades Hepáticas y Digestivas*	Spain	8	1.67
10th	*INSERM*	France	7	1.46

### Top ten funding agencies

A significant portion of the articles recovered, 62.8% of which had 301 publications and received financial support. Table 3 provides information on the top 10 funding agencies associated with NAFLD and its correlation with the gut microbiota from 2010 to 2022. These leading 10 agencies collectively contributed to 39.45% (*n* = 189) of the total published articles. In particular, the *National Natural Science Foundation of China* in China emerged as the most active funding agency in the field, supporting 24.63% (*n* = 118) of the articles. The *National Institute of Diabetes and Digestive and Kidney Diseases* in the USA (*n* = 23; 4.80%), the *National Institutes of Health* in the USA (*n* = 16; 3.34%) and the *National Key Research and Development Program of China* (*n* = 16; 3.34%) were followed closely.

**Table 3:** The top ten funding agencies with the most publications on the gut microbiota and NAFLD from 2010 to 2022

Ranking	Institute	Country	*n*	%
1st	*National Natural Science Foundation of China*	China	118	24.63
2nd	*National Institute of Diabetes and Digestive and Kidney Diseases*	USA	23	4.80
3rd	*National Institutes of Health*	USA	16	3.34
3rd	*National Key Research and Development Program of China*	China	16	3.34
5th	*Fundamental Research Funds for the Central Universities*	China	12	2.51
6th	*National Research Foundation of Korea*	South Korea	11	2.30
7th	*China Postdoctoral Science Foundation*	China	9	1.88
7th	*Instituto de Salud Carlos III*	Spain	9	1.88
9th	*Japan Society for the Promotion of Science*	Japan	8	1.67
9th	*National Institute on Alcohol Abuse and Alcoholism*	USA	8	1.67

### Top ten most active journals

According to the data presented in Table 4, the collective contribution of the top 10 journals/source titles constitutes approximately 24.21% of the general publications pertaining to research on NAFLD and its association with the gut microbiota. The *International Journal of Molecular Sciences*, which boasted an impact factor of 5.6 in 2023, exhibited the highest publication count, totaling 16 publications. Subsequently, *Nutrients*, with an impact factor of 5.9 in 2023, closely followed 14 publications, while *Frontiers in Microbiology*, featuring an impact factor of 5.2 in 2023, recorded 13 publications.

**Table 4:** List of the top 10 journals publishing research on gut microbiota and nonalcoholic fatty liver disease from 2010 to 2022

Ranking ^a^	Journal	Frequency	%	IF^b^
1st	*International Journal of Molecular Sciences*	16	3.34	5.6
2nd	*Nutrients*	14	2.92	5.9
3rd	*Frontiers in Microbiology*	13	2.71	5.2
4th	*Frontiers in Pharmacology*	10	2.09	5.6
4th	*Journal of Agricultural and Food Chemistry*	10	2.09	6.1
6th	*Scientific Reports*	9	1.88	4.6
7th	*Hepatology*	8	1.67	14
7th	*Journal of Clinical Hepatology*	8	1.67	NA
9th	*Biomedicine and Pharmacotherapy*	7	1.46	7.5
9th	*Food and Function*	7	1.46	6.1
9th	*Frontiers in Cellular and Infection Microbiology*	7	1.46	5.7
9th	*Modern Gastroenterology*	7	1.46	NA

^a^ Gap is left in the next ranking number when specific journals are given the same number

^b^ Impact factor (IF) based on Clarivate Analytics Journal Citation Reports (JCR) 2022

### Analysis of citations

By conducting a citation analysis, it was determined that the articles garnered an average of 43.23 citations, resulting in an h-index of 70 and a cumulative total of 20,705 citations. Among these articles, 122 did not receive any citations, while 124 obtained more than 100 citations. The citation count for these articles ranged from 0 to 1058. Table 5 shows the top ten publications on NAFLD and its correlation with diet, for a total of 5,637 citations. The citation range for these publications ranges from 387 to 882 [12, 14, 15, 66–72].

**Table 5:** Top-cited list of the top 10 most highly cited papers related to the gut microbiota and nonalcoholic fatty liver disease from 2010 to 2022

Authors	Title	Year	Source title	Cited by
Boursier et al. [66]	The severity of nonalcoholic fatty liver disease is associated with gut dysbiosis and shift in the metabolic function of the gut microbiota	2016	*Hepatology*	882
Le et al. [69]	Intestinal microbiota determines development of non-alcoholic fatty liver disease in mice	2013	*Gut*	681
Leung et al. [12]	The role of the gut microbiota in NAFLD	2016	*Nature Reviews Gastroenterology and Hepatology*	634
Loomba et al. [70]	Gut Microbiome-Based Metagenomic Signature for Non-invasive Detection of Advanced Fibrosis in Human Nonalcoholic Fatty Liver Disease	2017	*Cell Metabolism*	614
Mouzaki et al. [71]	Intestinal microbiota in patients with nonalcoholic fatty liver disease	2013	*Hepatology*	572
Raman et al. [72]	Fecal microbiome and volatile organic compound metabolome in obese humans with nonalcoholic fatty liver disease	2013	*Clinical Gastroenterology and Hepatology*	516
Del Chierico et al. [67]	Gut microbiota profiling of pediatric nonalcoholic fatty liver disease and obese patients unveiled by an integrated meta-omics-based approach	2017	*Hepatology*	467
Aron-Wisnewsky et al. [14]	Gut microbiota and human NAFLD: disentangling microbial signatures from metabolic disorders	2020	*Nature Reviews Gastroenterology and Hepatology*	456
Jiang et al. [68]	Dysbiosis gut microbiota associated with inflammation and impaired mucosal immune function in intestine of humans with non-alcoholic fatty liver disease	2015	*Scientific Reports*	428
Abu-Shanab and Quigley [15]	The role of the gut microbiota in nonalcoholic fatty liver disease	2010	*Nature Reviews Gastroenterology and Hepatology*	387

### Hot spots related to NAFLD and gut microbiota research

**Fig. 3:**
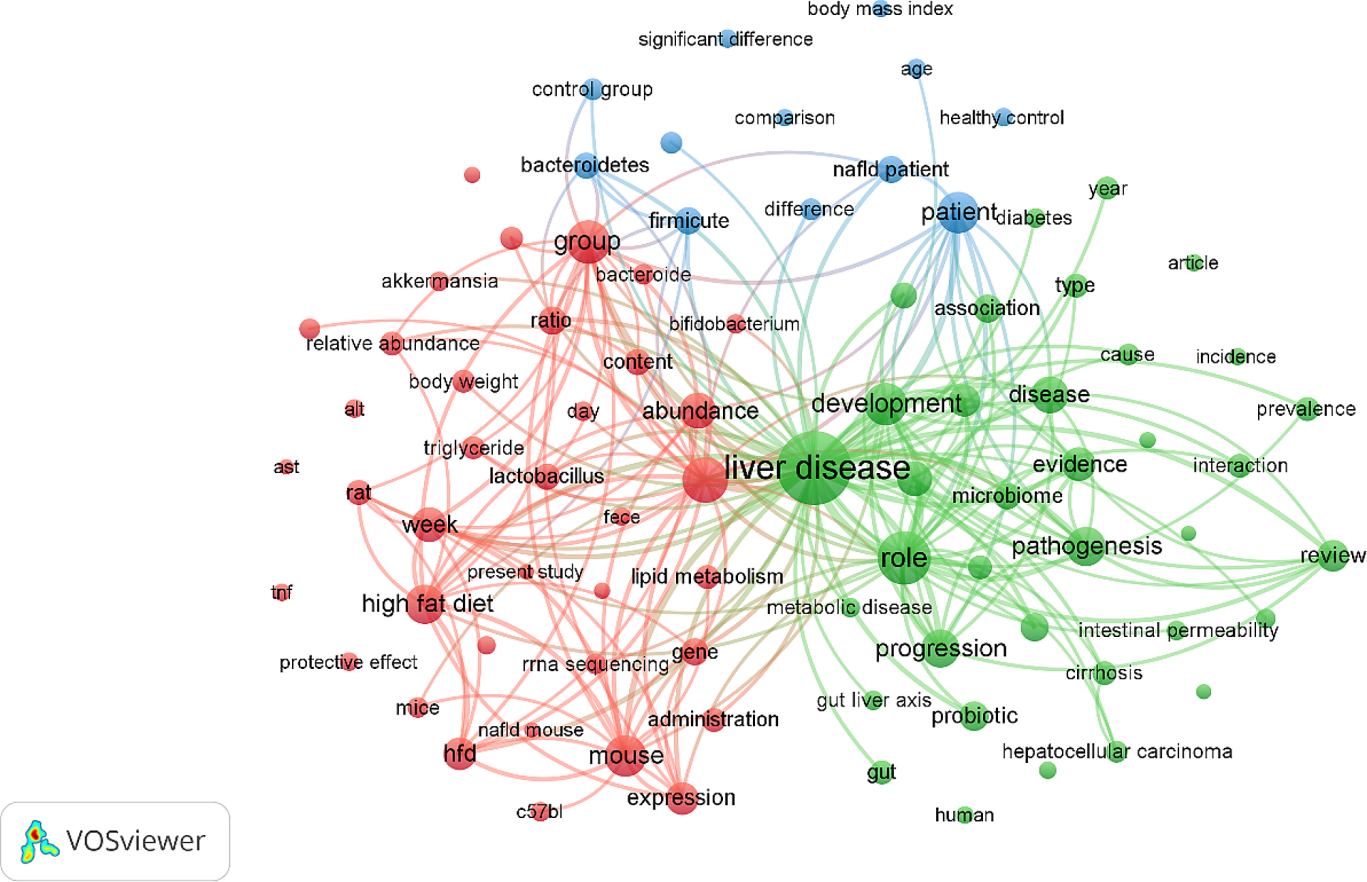
Network visualization map of terms in the title/abstract of publications related to the gut microbiota and nonalcoholic fatty liver disease from 2010 to 2022. The map was created using VOSviewer software version 1.6.20, with a minimum-term occurrence threshold of 20. Of the total 9,440 terms in this field, 142 terms reached this threshold and were divided into three clusters, each represented by a different color. The size of each node in the map indicates the frequency of a term’s usage across publications. The map was generated using VOSviewer software version 1.6.20

Figure 3 illustrates the main focal points concerning NAFLD and its connection to the gut microbiota from 2010 to 2022. Using VOSviewer analysis of the 479 retrieved documents, the titles and abstracts were searched for terms, resulting in the creation of a map featuring 142 terms. These terms were drawn from a total of 9,440 terms in the field and organized into three groups, each with a minimum of 20 appearances per term. The noteworthy terms on the map encompass (a) the impact of high-fat diets on the gut microbiome and its association with the development of NAFLD (red cluster); (b) the role of the gut microbiota in obesity and the development of NAFLD (blue cluster); and (c) the involvement of the gut–liver axis in the dysbiosis of the gut microbiome linked to NAFLD (green cluster).

### Future research direction analysis

**Fig. 4:**
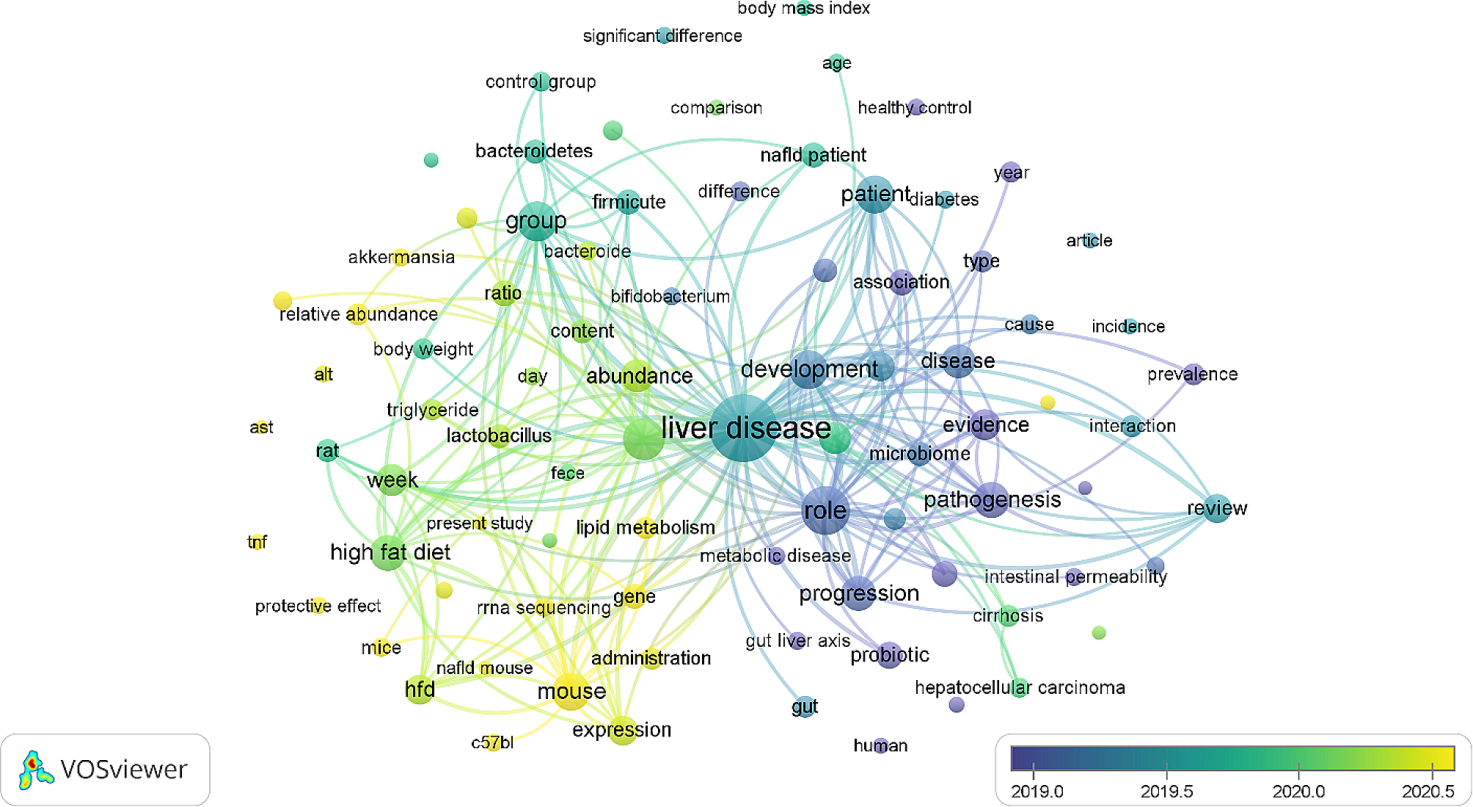
Visualization of Term Analysis of the Gut Microbiota and Nonalcoholic Fatty Liver Disease Publications (2010–2022). This network visualization map illustrates the analysis of terms found in the titles and abstracts of publications related to the gut microbiota and nonalcoholic fatty liver disease. The map shows the frequency of term appearances, with earlier occurrences represented in blue and later occurrences in yellow. The map was generated using VOSviewer software version 1.6.20

In Fig. 4, VOSviewer assigned distinct colors to each term based on its average frequency in all retrieved publications. The color scheme signifies the chronological distribution of term appearances, with blue indicating earlier occurrences and yellow representing more recent occurrences. Prior to 2020, the primary focus in this field revolved around ‘investigating the role of the gut microbiota in obesity and the development of NAFLD’ and ‘investigating the involvement of the gut–liver axis in the dysbiosis of the gut microbiota linked to NAFLD’. However, the term “impact of high-fat diets on the gut microbiome and its association with NAFLD development” emerged more recently after 2020, indicating the current trajectory of research interest.

## Discussion

This study represents the first in-depth bibliometric analysis focusing on global research trends in the association between the gut microbiota and NAFLD. It encompasses a variety of dimensions, such as types of documents, annual publication trends, contributions from top countries and institutions, leading journals with their impact factors, most cited articles, and a co-occurrence analysis of frequently used terms to pinpoint the most researched topics within this field.

The study’s results reveal a significant increase in research output, especially in the past decade. This escalation is evident in the exponential rise in publications, particularly from 2017 onward, indicating heightened interest and focus in the field. This pattern of intensification is likely a product of the expanding interest in the relationship between the gut microbiota and NAFLD, a relationship that is now acknowledged as essential in the holistic understanding of this condition [11–14, 66, 70].

The number of publications initially experienced a modest yet steady rise in output. Although the volume was limited, these publications played a significant role in garnering interest in the topic. For instance, an article by Jiang, W. et al. described the relationship between the gut microbiota composition and the development of NAFLD. Dysbiosis contributes to inflammation and impaired mucosal immune function [12, 15, 73]. Another study by Leung, C. et al. explored the link between dietary fats and the gut microbiota, illustrating the link between poor diet and consequent obesity, hepatic steatosis and NAFLD. Moreover, further studies have explored various aspects of NAFLD and the gut microbiota, including Abu-Shanab, A. et al.‘s research on identifying microbial metabolites as potential early indicators of NAFLD pathogenesis and Alkhouri N. and team’s work on pediatric NAFLD, which led to the development of novel histological scores to enhance the understanding and treatment of NAFLD in children [12, 15, 73].

In the second phase, from 2017 onward, there was more significant growth in research output. driven by enhanced microbiological techniques and a deeper understanding of the gut–liver axis. Studies have demonstrated that gut microbiota dysbiosis is closely linked to NAFLD in individuals with metabolic diseases such as obesity and type 2 diabetes. This period saw significant advances in identifying specific microbiome signatures that distinguish healthy individuals from those with NAFLD, despite challenges in differentiating these signatures from underlying metabolic disorders [14, 16]. Notably, pediatric NAFLD has also garnered attention, as metagenomics and metabolomics have revealed distinct microbial and compound alterations [67]. A major achievement was the development of a gut microbiota-derived metagenomic signature for predicting advanced fibrosis in NAFLD patients, revealing a noninvasive method for detecting advanced stages of the disease with high accuracy [70].

The geographical distribution of publications reveals a significant concentration of research activity in specific countries. China leads, followed by the United States, France, and Italy. This distribution highlights not only the global nature of NAFLD research but also the varying levels of interest and investment in different regions. China’s lead in publication output could be linked to its ongoing efforts to bolster scientific research, in addition to its widely known focus on traditional and integrative medicine approaches for metabolic diseases [74–77]. One such effort is China’s substantial investment in establishing state-of-the-art laboratories and research institutes to attract scientists and increase its research output [78, 79]. Another way that China is driving the observed increase in research output is establishing funding agencies.

Funding agencies have been demonstrated to play a pivotal role in shaping the focus and scope of scientific research [80]. An illustrative case of this influence, potentially contributing to China’s excellence in researching the gut microbiota and NAFLD, is the National Natural Science Foundation of China (NSFC). By serving as a major sponsor for both fundamental and applied scientific research, the NSFC has significantly improved China’s research outcomes. This is particularly evident in the substantial surge in publications following the establishment of a specialized NSFC dedicated to investigating the gut-liver axis [81, 82]. Nevertheless, the United States is not far behind; its substantial contributions are likely a consequence of its prominence in biomedical research and the availability of funding for such studies [83, 84]. The role of funding agencies is also noteworthy in the United States. Entities such as the National Institute of Diabetes and Digestive and Kidney Diseases and the National Institutes of Health (NIH) have been instrumental in advancing overall research in the United States, specifically concerning the gut microbiota [85]. This is exemplified in the NIH’s Human Microbiome Project [86] and various other projects [85].

Research on the gut microbiota and NAFLD has historically made meaningful contributions to our understanding of this relationship. In addition to receiving a high number of citations and demonstrating high h-index values, this research has been concentrated in high-impact journals such as the International Journal of Molecular Sciences, Nutrients, and *Frontiers in Microbiology*. These metrics, along with acceptance in high-impact journals and subsequent publications, underscore the significance of these works and their recognition in the field [87].

The term co-occurrence analysis revealed the varied nature of research in this area over the past decades. Initially, interest focused on understanding the relationship between the gut microbiome and liver health, as highlighted in topics such as “investigation into the role of gut microbiota in obesity and the development of NAFLD” and “the involvement of the gut-liver axis in gut microbiome dysbiosis linked to NAFLD” [88]. More recently, in parallel with the global rise in dietary-related health issues and the need to understand how lifestyle influences disease etiology, research has shifted its focus to understanding the impact of high-fat diets on the gut microbiota and their link to NAFLD development [89–93].

### Future directions

In the future, research on the gut microbiota and NAFLD may focus more on a deeper understanding of how diet, gut bacteria, and liver health are connected. This could involve studying how specific foods affect the microbiota and, in turn, NAFLD. Researchers should also focus on how lifestyle factors such as exercise and daily routines influence the gut-liver axis. Advanced genetic and metabolic technology should open up new avenues to diagnose or treat NAFLD by studying how gut microbes interact with human cells.

Another promising direction for managing NAFLD is personalized medicine. Given the variability in gut microbiota composition among individuals, personalized dietary or probiotic interventions based on one’s specific microbiome profile could be explored. This approach would require a concerted effort in large-scale data collection and analysis, integrating microbiome data with genetic, metabolic, and clinical information. Furthermore, the interaction between the gut microbiota and other organ systems beyond the liver is likely to become a significant area of study, offering an understanding of the systemic nature of NAFLD and its links with other diseases, such as cardiovascular diseases and type 2 diabetes.

### Limitations

Several limitations of our study should be acknowledged. First, our reliance on Scopus as the sole data source, while advantageous due to its extensive database of peer-reviewed literature, may have led to the exclusion of relevant studies not indexed in this platform. While Scopus’s comprehensive coverage mitigates this limitation to some extent, there is still a possibility of missing pertinent literature from other databases. Second, the methodology focused on identifying relevant terms in the titles and abstracts of publications and missing studies that discussed the gut microbiota-NAFLD relationship in different parts of the literature. Third, determining the geographic origin of research based on affiliation information may not always be accurate. Fourth, the analysis focused specifically on the connection between the gut microbiota and NAFLD incidence, excluding other potential factors, such as genetics, lifestyle, or the environment, that could also be important in NAFLD development. Fifth, we considered only English-language publications. Sixth, we prioritized established terms such as NAFLD in our literature search to capture the research trends reflecting our specific interest in NAFLD itself rather than encompassing broader related terminology. Although this approach yielded a robust collection of existing studies, some of the latest publications that encompass both NAFLD and the newer term MAFLD might have been missed. Finally, our study was primarily descriptive, aiming to identify trends and patterns in the literature rather than evaluating the quality, impact, or relevance of individual studies in the broader context of NAFLD research.

## Conclusions

This study mapped the publication output of gut microbiota and NAFLD research. There has been significant growth in publications since 2017. Key findings include the importance of gut microbiota imbalances in NAFLD and the impact of diet. The research is global, with China and the U.S. making major contributions. In the future, research should explore diet, lifestyle, and the gut-liver connection. Advanced technology should lead to breakthroughs in personalized medicine and a deeper understanding of the wider effects of NAFLD.

## Data Availability

All the information produced or examined in this research is contained within this published article. Additional datasets utilized in the course of this study can be obtained from the corresponding authors.

## Abbreviations

IF: Impact factor
MeSH: Medical Subject Headings
NAFLD: Nonalcoholic fatty liver disease
NIH: National Institutes of Health
NSFC: National Natural Science Foundation of China
p: Statistically significant
R^2^: Coefficient of determination in linear regression
r: Correlation coefficient
Scopus: SciVerse Scopus database
USA: United States of America
